# KSHV encoded miRNA single nucleotide polymorphisms identified in clinical samples can affect miRNA processing and level of expression

**DOI:** 10.1186/1750-9378-7-S1-P40

**Published:** 2012-04-19

**Authors:** Vickie Marshall, Soo-Jin Han, Eugene Barsov, Octavio Quinones, Alex Ray, Robert Yarchoan, David Ott, Rolf Renne, Denise Whitby

**Affiliations:** 1 Viral Oncology Section, AIDS and Cancer Virus Program, SAIC-Frederick, National Cancer Institute, National Institutes of Health, Frederick, MD, USA; 2 Department of Molecular Genetics and Microbiology, University of Florida, Gainesville, FL, USA; 3 Retrovirus Assembly Section, AIDS and Cancer Virus Program, SAIC-Frederick, Frederick, MD, USA; 4 Data Management Services, Inc., NCI-Frederick, Frederick, MD, USA; 5 HIV and AIDS Malignancy Branch, National Cancer Institute, National Institutes of Health, Bethesda, MD, USA

## 

MiRNAs are a class of non-coding RNA molecules between 19-25 nucleotides in length that have been shown to be involved in many biological processes by post-transcriptionally regulating gene expression. Aberrant miRNA expression has recently been associated with disease including many human cancers. Kaposi’s sarcoma associated herpesvirus (KSHV) encodes 12 miRNAs located within the latency associated region. We previously reported single nucleotide polymorphisms (SNP) in the sequence of KSHV encoded mature and pre-miRNAs from clinical samples [1]. An earlier report showed that a SNP in mir-K12-5 resulted in increased expression of the mature miRNA [2]. In the current study, we have analyzed three different classes of miRNA polymorphisms to determine if any affect mature miRNA processing and expression. The identified SNPs include single and multiple polymorphisms within the pre-miRNA transcript, single mutations within the terminal loop, and single sequence changes within the mature miRNA.

We used four complimentary approaches to detect differences in miRNA processing and expression resulting from sequence polymorphisms. Analysis of KSHV miRNA expression levels in PEL cell lines using custom ABI real time qPCR assays showed differential expression that correlates with sequence variation. Lentiviral vectors constructed to express wild type and variant pre-miRNAs were transduced into 293T cells to make stably expressing cell lines. miRNA expression was assessed using custom ABI real time qPCR assays. Luciferase reporter assays were performed following transient transfections of each miRNA. In addition, *in vitro* maturation assays were performed to assess differences in Drosha/DGCR8 and Dicer cleavage between wild type and variant pre-miRNAs.

Our results show that polymorphisms within the pre-miRNA sequence can cause subtle expression differences as in the case of KSHV miR-K12-6 or more profound changes as observed in miR-K12-5. This is also the case with SNPs located within the terminal loop as some miRNAs exhibited no discernable change, miR-K12-7 and miR-K12-10, while others can cause a reduction in mature miRNA transcripts as noted in miR-K12-4. Mutations within the mature transcript appear to have the most potential to effect mature transcript processing and expression. The polymorphism within miR-K12-2 results in reduction of mature miRNA levels while the multiple changes in miR-K12-9 leads to complete loss of the mature transcript.

Our data clearly show that SNPs can affect pre-miRNA processing resulting in changes in mature miRNA expression levels. The biological significance of these phenotypic and genotypic variants merits further study.
